# Reduced M2 macrophages and adventitia collagen dampen the structural integrity of blood blister–like aneurysms and induce preoperative rerupture

**DOI:** 10.1111/cpr.13175

**Published:** 2021-12-30

**Authors:** Dingke Wen, Ruiqi Chen, Hao Li, Jun Zheng, Wei Fu, Ziyan Shi, Chao You, Mu Yang, Lu Ma

**Affiliations:** ^1^ Department of Neurosurgery West China Hospital Sichuan University Chengdu Sichuan China; ^2^ Department of Neurology West China Hospital Sichuan University Chengdu Sichuan China; ^3^ Translational Centre for Oncoimmunology Sichuan Cancer Hospital & Institute University of Electronic and Science Technology of China Chengdu China

**Keywords:** aneurysm rerupture, blood blister–like aneurysm, intracranial aneurysm, macrophage polarization, pathology

## Abstract

**Objective:**

Blood blister–like aneurysms (BBAs) are extremely rare aneurysms. They are predisposed to preoperative rerupture with a high case‐fatality rate. Here, we attempt to interrogate the distinct clinicopathology and the histological basis underlying its clinical rerupture.

**Methods:**

Three middle meningeal arteries, 11 BBA (5 reruptured, 6 non‐rerupture) and 19 saccular aneurysm samples were obtained for histopathological investigation. Three reruptured BBAs, 3 non‐reruptured BBAs and 6 saccular (3 ruptured, 3 unruptured) aneurysms were obtained for quantitative flow cytometry analysis.

**Results:**

Compared with true saccular aneurysms, the BBA aneurysm wall lacks arterial stroma cells including CD31+ endothelial cells and α‐SMA + smooth muscle cells. Only fibroblasts and adventitial collagen were observed in the BBA aneurysm wall. Meanwhile, BBAs were enriched with infiltrated inflammatory cells, especially polarized macrophages. Based on the rerupture status, those reruptured BBAs showed drastically reduced fibroblasts and adventitia collagen. Moreover, M2‐polarized macrophages were observed dominant in BBAs and exhibit repairing cellular functions based on their interplays with arterial fibroblasts. Reduced M2 macrophages and arterial tissue repairing modulation may be responsible for the decreasing collagen synthesis and fibrosis repairment, which potentially dampens the aneurysm integrity and induces BBA aneurysm reruputre.

**Conclusions:**

BBAs poses histopathological features of occult pseudoaneurysms or dissecting aneurysms. Reduced M2 macrophages and adventitia collagen may dampen the structural integrity of BBAs and induce preoperative rerupture.

## Graphical abstract

The BBAs with decreased M2 macrophages and adventitia collagen possess risk of imminent aneurysm rerupture. Targeting M2‐polarized macrophages and its interplay with arterial fibroblasts may be a therapeutic potential in BBAs.

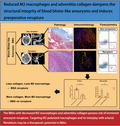

## 1 INTRODUCTION

Blood blister–like aneurysm (BBA) covers a rare but treacherous subtype of intracranial aneurysms that typically grow on the non‐branching sites of internal carotid arteries (ICA).^1^, ^2^ They were first introduced as BBAs (or BBAs) in 1998 by Japan physician Ishikawa based on the blister‐like appearance upon autopsy.^1^ Features of BBAs have been subsequently summarized in literature, including broad neck, supra‐clinoidal position and prevalence on right side and in young females.^3^, ^4^, ^5^ Despite occasional case reports documenting their distinct aneurysm structure, the pathophysiology and aetiology remain elusive.^1^, ^6^

In contrast to the rare occurrence of approximately 1% in all ruptured brain aneurysms, BBAs exhibit an unproportionally high case‐fatality rate.^1^, ^2^, ^5^, ^6^, ^7^ The main contributor was thought to be the unexpected preoperative rerupture during rapid progression.^8^, ^9^, ^10^, ^11^ Aneurysm rerupture, regardless of aneurysm subtype, can result in lethal consequences such as conscious deterioration and accumulation of subarachnoid haemorrhage (SAH). Additionally, it is also strongly associated with poor outcomes and increased risk of peri‐operative complications such as aneurysm avulsion, parent artery laceration and uncontrollable intraoperative bleeding. Hence, BBAs have been reckoned as an unneglectable risk in cerebrovascular neurosurgery.^2^

Previous studies by Aoki, Frosen and Lawton have meticulously demonstrated the pathology of conventional intracranial saccular aneurysm.^12^ The diverse biological functions of smooth muscle cells and macrophages have been proven pivotal throughout the aneurysm development. However, owning to the limited attention given to BBAs, it remains unknown whether, or to what extent, could similar pathological pattern be applied in BBAs. The clear pathological definition of BBAs remains controversial. Recently, we have described the pathological drives for the progression phenomenon in BBA.^3^, ^13^ Intriguingly, the initial results indicate significant discrepancies in pathology and cellular interplay between BBAs and saccular aneurysms. Likewise, irrespective of the aneurysm size, whether the rerupture of BBAs is associated with certain vasculopathy was poorly understood. Therefore, our current study aimed to illustrate the pathological features of BBAs and reveal histological basis for BBA preoperative rerupture. Relevant intercellular interplays and molecular changes were also investigated.

## 2 METHODS

### 2.1 Patient inclusion and exclusion

From 2017 to 2020, a total of 7,498 patients with intracranial aneurysms were screened for inclusion in our neurological centre. Detailed inclusion criteria have been described in our previous publications.^3^ Briefly, patients were included as BBAs if (1) the aneurysm(s) was(were) located on the non‐branching sites on the dorsal (most commonly), dorsal lateral or ventral part of internal carotid artery and (2) the aneurysms had no clear relation with adjacent arterial bifurcation. Exclusion criteria were as follows: (1) Digital subtraction angiography (DSA) confirmed posterior communicating artery aneurysm with haemodynamic relationship to the adjacent posterior communicating artery origin, (2) DSA‐confirmed anterior choroidal aneurysms, and (3) infundibulum expansion adjacent to posterior communicating artery origin.

### 2.2 Clinical information

For baseline comparison, clinical information was collected from hospital imaging system, laboratory system and history information system. Patients with BBAs were categorized into reruptured group and non‐reruptured group according to the in‐hospital clinical records. BBAs were considered as reruptured BBAs if patients showed CT‐confirmed re‐haemorrhage with/without clinical deterioration during the interval between initial subarachnoid haemorrhage and surgical intervention. BBAs were included as non‐rerupture BBAs if patients were confirmed with stable during pre‐operational observation and no CT angiography (CTA) or DSA‐confirmed aneurysm enlargement was documented.

### 2.3 Histopathological assays

Based on the included BBA patient, we obtained 11 BBA samples. In comparison, 19 saccular aneurysms samples and 3 middle meningeal arteries were obtained from the bio‐database of West China hospital cerebrovascular neurosurgery sample bank, detailed demographic information was included in Table S1. To obtain saccular aneurysm, the aneurysm dome was carefully dissected after satisfactory placement at the aneurysm neck. In BBA cases, we first reaffirm the accurate diagnosis during gross inspection. Then, we would proceed to collect the BBAs sample when aneurysm shows collectable fibrous dome after a mini‐curved clip placed at the neck. Micro‐scissors were used to dissect the remnant aneurysm dome along the blade of clips as the premise of stable clipping.^3^

Cryosection slides and paraffin‐embedded blocks were used for histopathological studies. H&E and Masson's trichrome were used for basic pathological staining. Collagen content was calculated by ImageJ as the positive area percentage under 20× magnification. Anti‐CD31, anti‐α‐SMA, anti‐vimentin antibody were used to detect vascular epithelial cells, smooth muscle cells and adventitia fibroblasts, respectively. Anti‐CD45 antibody was used to detect myeloid‐derived infiltrated inflammatory cells. Iba1 was used to identify macrophages. Caspase‐3 was used to detect the inflammatory apoptosis level in aneurysm wall. Matrix metalloproteinase 9 (MMP‐9) was also used to evaluate the pro‐inflammatory functions in BBAs. On the other hand, vascular endothelial growth factor (VEGF) was used to detect the tissue repairing process in aneurysm wall. Inducible nitric oxidate synthase (iNOS) and Arginase 1 were used to clarify the functional polarization of in situ macrophages, which also suggest the pro‐inflammatory and anti‐inflammatory functions of macrophages, respectively. Antibody used in this study are included in Table S2.

### 2.4 Flow cytometry assays

Tissue flow cytometry was used to analyse the overall macrophage polarization status in the BBAs. Six freshly collected BBA samples (3 from berrylike BBAs and 3 non‐berrylike BBAs) and 6 conventional saccular aneurysms (3 ruptured and 3 unruptured) were prospectively collected for tissue flow cytometry experiments. Fresh aneurysm tissue was collected from the operation room and transported in 4℃ to the laboratory for flow cytometry. Aneurysm tissue was dissociated in digestion buffer (digestion buffer = 0.25% trypsin + 2.5 mg/ml collagenase + 50 U/ml DNase; products were purchased from Sigma‐Aldrich) into single cell for antibody staining. Red blood cells were lysed by ACK buffer (Sigma‐Aldrich) prior to antibody staining. Flowjo 10.0 was used for flow cytometry analysis and gating. CD45, CD11b, CD86 and CD163 were used separately for the identification of macrophage phenotypes. Detailed antibody information was enclosed in Table S2.

### 2.5 Statistics

*t* Tests, chi‐square tests as well as Fisher's exact tests were used to test the statistical significance between/among groups. SPSS 26 (IBM™ SPSS 26) was used for statistical analysis, and GraphPad Prism 8 (GraphPad Software, LLC) was used for statistical figures for illustration purposes. In univariate analysis, factor showing *p* value <0.05 was considered clinically significant. Other analysis that showed a *p* value <0.05 was counted as statistically different. This study was approved by the West China Hospital of Sichuan University Biomedical Research Ethics Committee, Institutional Review Board, and was performed in compliance with the institutional regulations. All patients’ informed consent forms have been received.

## 3 RESULTS

### 3.1 Unique histopathological changes of BBAs compared with normal arteries and saccular aneurysms

**FIGURE 1:**
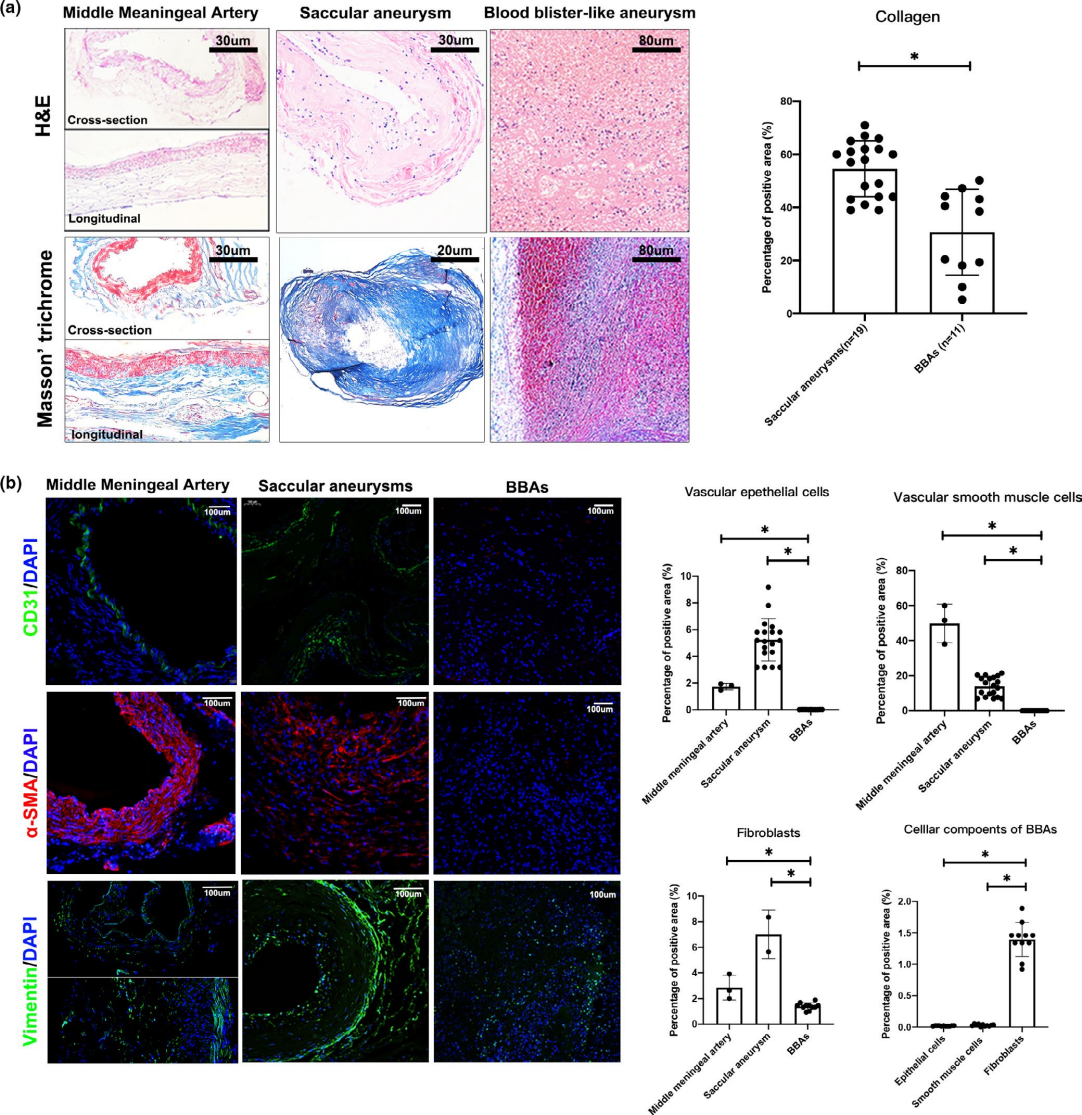
Histopathological study of blood blister–like aneurysms (BBAs). Pathological comparison among healthy middle meningeal arteries, conventional saccular aneurysms, and BBAs in H&E and Masson's trichrome stain (a, left panel). Collagen competent was significantly reduced in BBAs when compared to true saccular aneurysms (a, right histogram) (30.61% ± 4.88% vs. 54.53% ± 5.62%, *p *< 0.01). Immunofluorescence stain of arterial cells marked by CD31 (vascular endothelial cells, EC), α‐SMA (vascular smooth muscle cells, SMC) and vimentin (fibroblasts, FB) in healthy arteries, saccular aneurysms and BBAs (b, left panel). Results showing saccular aneurysms with reduced smooth muscle cells, but elevated endothelial cells and fibroblasts (All *p *< 0.05). All cell types are reduced in BBAs (0.015% EC, <0.01% SMC and 1.40% FB). Only some residual fibroblasts were observed (b, right panel). All scale bars are specified in the figures

To dissect the histopathology basis of BBAs, a sequelae of histopathological research were performed (Figure 1). H&E and Masson's middle meningeal artery showed regular pathological baseline of normal artery (Figure 1a). Results of normal middle meningeal arteries exhibited good lineage of endothelial cells in the tunica intima, streamlined smooth muscle cells in tunica intima and loose connective collagen that consists mainly fibroblasts in adventitia (Figure 1a). While saccular aneurysm lost most endothelial cells in the intima, the smooth muscle cells showed irregular and disoriented proliferation in the intima. Connective collagen in the adventitia proliferated densely and intertwined with the tunica media (Figure 1a). Conversely, the main structure of BBAs was luminal thrombi wrapped by a thin collagenous adventitia, while features of chronic degenerative changes were rarely observed (Figure 1a). Masson's trichome showed the average collagen fraction of BBAs was significantly reduced compared with that in the conventional saccular aneurysms (30.61% ± 4.88% vs. 54.53% ± 5.62%, *p *< 0.01; Figure 1a). Immunohistology stains of CD31, α‐SMA and Vimentin confirmed the observation in H&E stain. Comparative analysis of immunofluorescence showed that only fibroblasts in the adventitia retained in BBA (1.40%), while endothelial cells (0.015%) and smooth muscle cells (<0.01%) were both almost absent (Figure 1b). These results indicated that the BBAs, regardless of their various presentations, exhibit typical pseudoaneurysm feature that is distinct from conventional saccular aneurysms.

### 3.2 Pathological comparison between reruptured BBAs and non‐rerupture BBAs.

**FIGURE 2:**
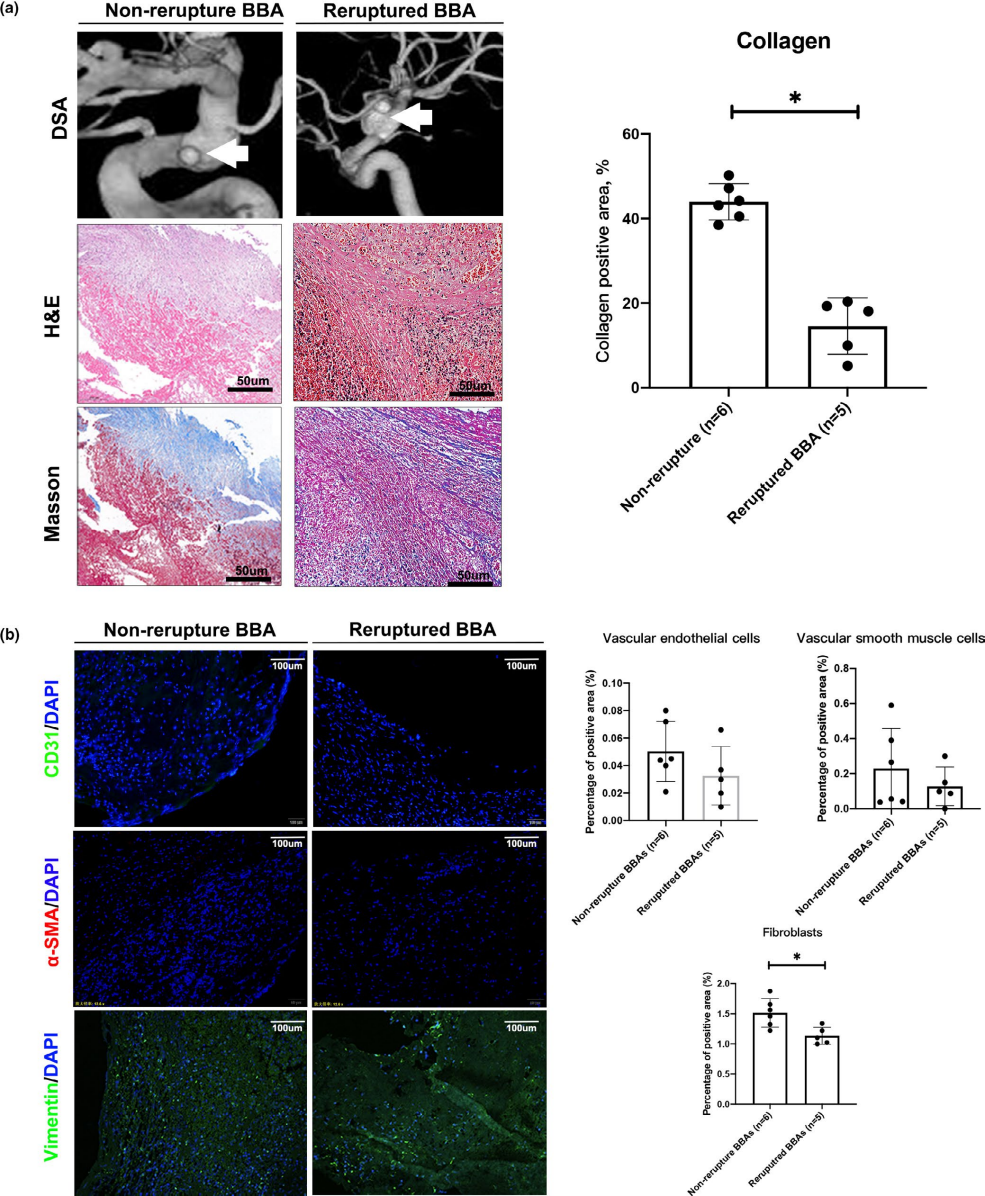
Histopathological study of non‐rerupture and reruptured BBAs. Histological study indicated that the collagen level is significantly reduced in reruptured BBAs (14.6% ± 6.64% vs. 43.95% ± 4.29%, *p *< 0.05) (a). No significantly differences were observed between non‐rerupture BBAs and reruptured BBAs in vascular endothelial cells, smooth muscle cells (EC: 0.05% ± 0.021% vs. 0.032% ± 0.019%, *p *= 0.208; SMC: 0.23% ± 0.2% vs. 0.12% ± 0.11%, *p *= 0.382). Fibroblasts are observed more in the non‐rerupture BBAs (1.517% ± 0.23% vs. 1.136% ± 0.14%, *p *= 0.018) (b). All scale bars are specified in the figure

To explore the histological nuances underlying distinct BBA presentations. We regrouped the patients according to the rerupture history. Five BBAs presented with in‐hospital rerupture were included in rerupture group, while the rest 6 BBAs were considered non‐rerupture BBAs. For the 5 reruptured BBAs, the collagen fraction was significantly lower than that in the non‐reruptured BBAs (14.6% ± 6.64% vs. 43.95% ± 4.29%, *p *< 0.05; Figure 2a). Correspondingly, more fibroblasts are observed in the non‐rerupture BBAs (1.517% ± 0.23% vs. 1.136% ± 0.14%, *p *= 0.018; Figure 2b). No significant differences were observed between non‐rerupture BBAs and reruptured BBAs in vascular endothelial cells and smooth muscle cells (vascular endothelial cells: 0.05% ± 0.021% vs. 0.032% ± 0.019%, *p *= 0.208; smooth muscle cells: 0.23% ± 0.2% vs. 0.12% ± 0.11%, *p *= 0.382; Figure 2b). These results suggest that the progression and rerupture of BBA may not follow the conventional vascular pathological patterns in saccular aneurysms. Instead, they may be associated with arterial destruction with fibrous collagen repairment, which is mainly mediated by fibroblasts rather than endothelial cells and smooth muscle cells.

### 3.3 Diverse infiltrated macrophage functions underlie different aneurysm pathophysiology

**FIGURE 3:**
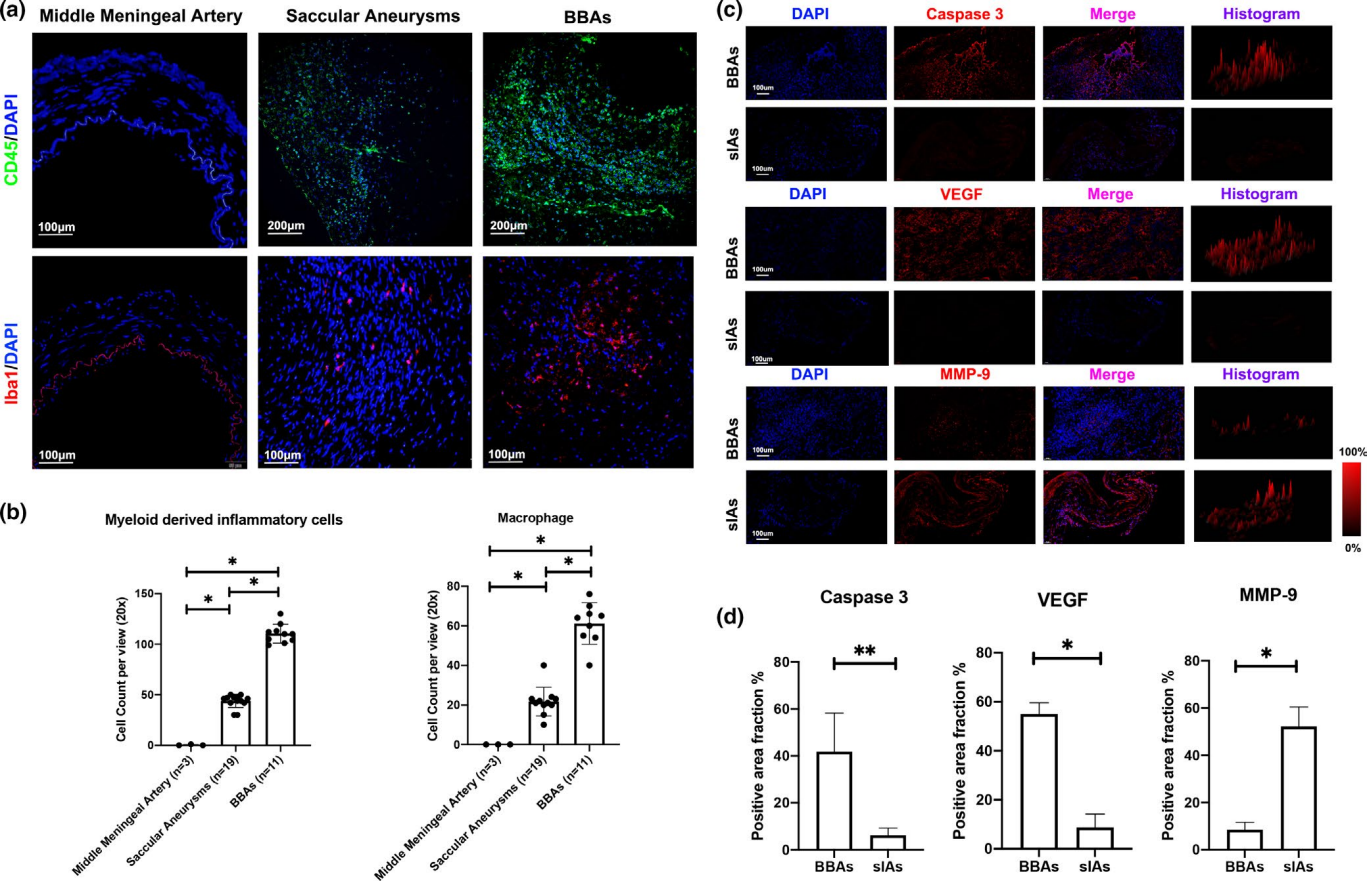
Infiltrated inflammatory cell in BBAs and saccular aneurysms. Compared with healthy arteries and saccular aneurysms, blood blister–like aneurysms showed elevated myeloid‐derived inflammatory cells level in the aneurysm wall (a), macrophages were the major component among inflammatory cells. The difference was statistically significant (b). Tissue apoptosis, pro‐inflammatory cytokines as well as tissue repairing cytokines were measured by the level of caspase‐3, matrix metalloproteinase 9 and vascular endothelial growth factor (VEGF) (c and d). Scale bars are specified in figures, **p < 0.01, *p < 0.05

Based on the pathological findings, we speculated the infiltrated inflammatory cells, but not the arterial stroma cells, influence fibroblasts proliferation and collagen repair in BBAs. Immunofluorescence assays were then carried out to identify the inflammatory cells and functional heterogeneity. Other than arterial stroma cells, infiltrated inflammatory cells, mostly macrophages (stained by CD45 and IBA1), were observed in the BBA aneurysm wall (*p *< 0.05; Figure 3a, b). To further appreciate the biological divergence of the macrophages in conventional saccular aneurysms and BBAs, we performed quantitative immunofluorescence comparison between the secretory cytokines in the aneurysms. BBA tissue show increased caspase‐3 level compared with saccular aneurysms (*p *< 0.05; Figure 3c, d). VEGF was also increased in the BBAs compared with that in the saccular aneurysms (*p *< 0.05; Figure 3c, d). In contrast, the MMP‐9, a pro‐inflammatory extracellular lysis enzyme mainly produced by macrophages, was reduced in the BBAs compared with that in the saccular aneurysms (*p *< 0.05; Figure 3c, d). Interestingly, these data prompted that the macrophage, rather than other cell types, centres the pathophysiology of aneurysms collagen repair in BBAs. The cellular apoptosis and proliferative level in BBAs were increased, while pro‐inflammatory process in saccular aneurysms was more prominent. It strongly indicated a functional dichotomization in different aneurysm phenotypes.

### 3.4 Macrophage polarization in BBAs is associated with the rerupture

**FIGURE 4:**
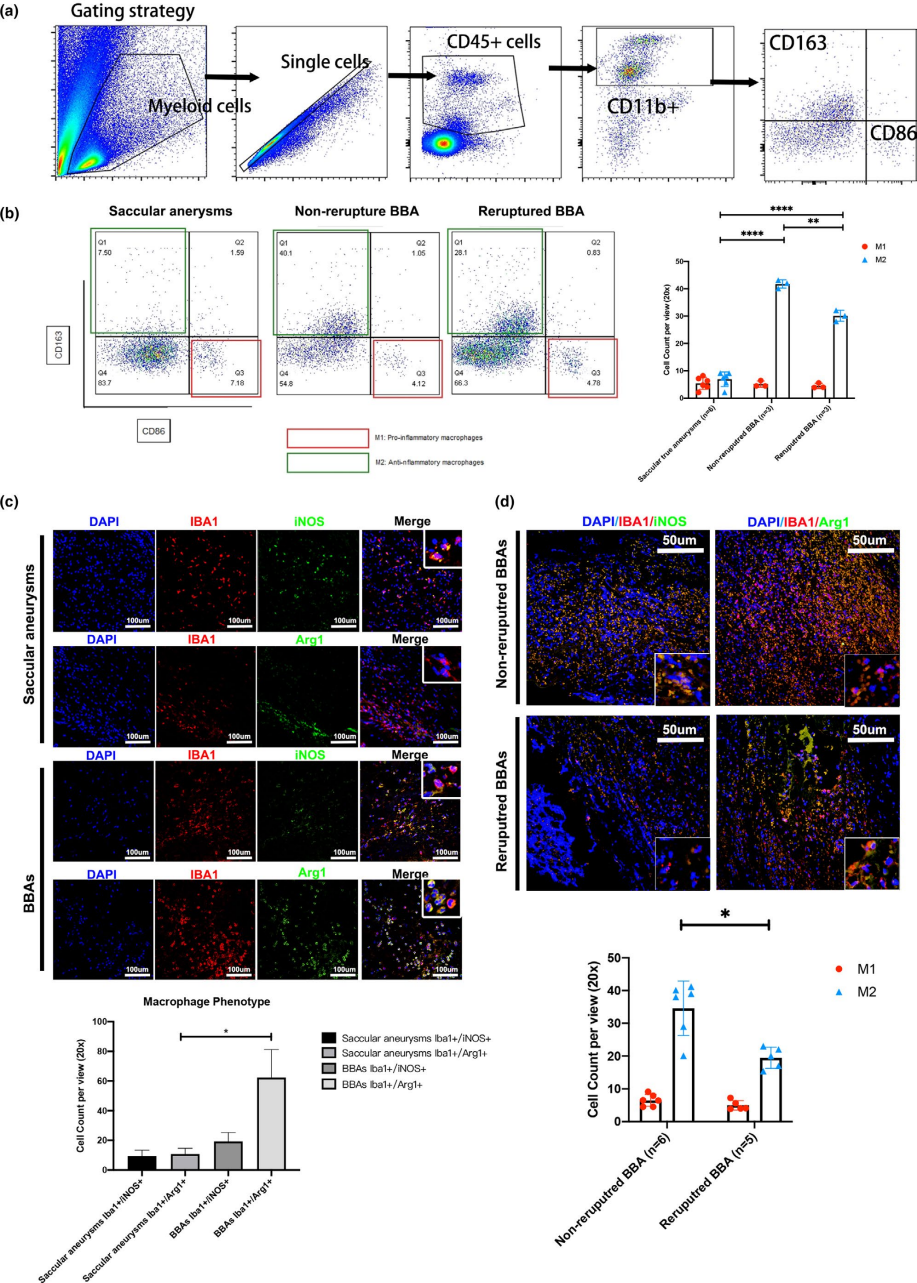
Identification of macrophage polarization status in BBAs with/without rerupture history. Flow cytometry gating strategy of aneurysm tissues (a). Flow cytometry indicates that the CD45+CD11b+CD163+ M2 macrophages are significantly elevated in BBAs compared with that in the saccular aneurysms (B), and reruptured BBAs show less increased M2 macrophages (b). Immunofluorescence stains validate that the functional IBA1+/Arg1+ M2 macrophages are also increased in BBAs (c). Reruptured BBAs showed reduced M2 macrophages compared with their counterparts without rerupture history (d). All scale bars are specified in the figures. ****p < 0.0001, *p < 0.05

Following, we set out to investigate what role did macrophage play in BBAs pathology and its preoperative rerupture. The function of macrophages is dependent on their polarizations. Pro‐inflammatory polarization M1 and anti‐inflammatory M2 (also known as alternative polarization or repairment polarization) are separately suggestive of distinctive macrophage functions. Therefore, we continued to explore whether macrophage polarization underlies clinical presentations. Macrophage polarization was determined phenotypically in flow cytometry and functionally in immunohistochemistry (Figure 4a).

Flow cytometry revealed anti‐inflammatory M2 (CD45^+^CD11b^+^CD163^+^) macrophages in BBAs are significantly enriched in numbers compared with both M1 (CD45^+^CD11b^+^CD86^+^) and M2 (CD45^+^CD11b^+^CD163^+^) macrophages in saccular aneurysms. The M2 in BBAs also outnumbered the M1 macrophages in BBAs in both reruptured and non‐rerupture BBAs (both *p* values <0.0001) (Figure 4b). Notably, non‐rerupture BBAs showed significantly more M2 macrophages compared than that in the reruptured BBAs (*p *= 0.0013) (Figure 4b). Immunofluorescence staining examined the results observed in flow cytometry, showing that the Arg1^+^/IBA1^+^ macrophage (M2) count is higher than the iNOS^+^/IBA1^+^ macrophages (M1) in BBAs compared with that in the conventional saccular true aneurysms (Figure 4c). M2 macrophages, however, is adversely reduced in reruptured BBAs when comparing to the compartments in non‐rerupture BBAs (*p *= 0.0169; Figure 4d). In all, flow cytometry and immunofluorescence assays together pointed out that the M2 macrophages are the essential polarization phenotype in BBAs compared with saccular aneurysms. M2 macrophages outnumber M1 in BBAs, especially in non‐reruptured BBAs, exhibiting robust repairment participation in the pathophysiology of non‐reruptured BBAs. Reduced M2 macrophages may be associated with severe destructed aneurysm wall and hint aneurysm structure deterioration.

## 4 DISCUSSIONS

In this study, by evaluating the clinicopathology of BBAs, we examined the potential pathophysiology under BBAs and their clinical rerupture. We noticed BBAs are typical pseudoaneurysms that are distinct from conventional saccular aneurysms. Based on the clinical presentation, reruptured BBAs showed drastically reduced fibroblasts and adventitia collagen. Moreover, macrophages, especially M2‐polarized phenotypes, were dominant in BBAs pathology and exhibit unique molecular functions based on their interplays with arterial cellular components. Reduced M2 macrophages and tissue repairing functions may be responsible for the decreasing collagen synthesis and fibrosis repairment, which potentially dampens the aneurysm integrity and induces aneurysm reruputre.

The aetiology and pathology of BBAs have remained controversial for decades.^1^, ^2^, ^14^, ^15^ Most studies focusing on BBAs were case reports that lack of proper comparison.^1^, ^10^, ^13^, ^16^ In‐depth histological investigations of aneurysm histopathology mostly excluded BBAs owning to the contentious aneurysm heterogeneity.^17^, ^18^, ^19^ By reporting this BBA pathology cohort, our current study provided histopathological evidence that BBAs may be a special type of pseudoaneurysm that lack normal atherosclerotic structure and stroma cells. The adventitia collagen wrapped thrombi resembled the structure of pseudoaneurysms or thrombotic dissecting aneurysms.^3^, ^20^, ^21^, ^22^ This explains the clinical observations that BBAs can be initial DSA‐negative or in an irregular shape, because limited lumen or atherosclerotic vasa vasorum enables contrast in‐flow at early arterial trauma.^22^ Whereas only in late stage when thrombi were formed that the contrast may diffuse in and reveal the aneurysm, various reasons to trigger BBA initiation are still in need for further investigation.

Owning to the unique pseudoaneurysm pathology, we reiterate our research focus on the collagenous adventitia instead of conventional endothelial cells and smooth muscle cells. Collagen adventitia has been proven a pivotal component of arteries and aneurysms, providing essential tensile strength, nutrients and inflammatory cell reservoir.^23^, ^24^ Fibroblasts are the main cell type that produce collagen fibres to maintain structure and wound healing.^23^ Under pathological conditions, arteries/aneurysms experience a dynamic and delicate balance of collagen synthesis and collagen degradation mediated by fibroblasts.^25^ Once the balance breaks, arterial lesion would prone to deteriorate or rupture.^17^, ^18^, ^19^ Our current finding implied that the reduced and destructed collagen is prominent in reruptured BBAs and directly leads to the instability of its structure. It emphasized the role of collagen components in maintaining aneurysm structure integrity and prompted a great possibility of taking it as a prediction biomarker for future study. Future collagen‐specific MRI may aid the evaluation of BBA rupture prediction.^26^

Additionally, the inflammatory process in BBAs was proven to be mediated by macrophages. Of note, these observations largely challenged the previous idea proposed by Ishikawa that BBAs are not involved with inflammatory responses. It is hence reasonable to consider BBAs as macrophage‐mediated inflammatory vasculopathies rather than simple blood thrombi. Previous research has proven that macrophage is a major mediator of arterial pathophysiology.^17^, ^27^, ^28^, ^29^, ^30^, ^31^ Nowiki's study signified that M1‐polarized macrophages are indispensable in the development of intracranial aneurysms owning to their iconic production of including tissue necrosis factor (TNF), Cx3Cr1 and iNOS.^32^ Others specified that M2 might participate in lesion fibrosis, granulations and wound healing by secreting extracellular matrix, VEGF and CCL‐18.^33^, ^34^, ^35^ Madsen's research also corroborated that M2‐like macrophages assist the removal and remodelling of collagen scaffold in tissue repairing by uptaking collagen debris.^36^ The expression of CD206 (Mannose receptor), CD163 as well as Arginase 1 in M2 macrophages indicated their function towards tissue repair and debris clearance.^37^, ^38^ In our study, the flow cytometry analysis showed that M2‐polarized macrophages were the leading inflammatory phenotype in BBAs, and this tissue repairing process was less significant in reruptured BBAs. Immunofluorescence further validated more CD163 and Arginase 1 expression on M2 macrophages in non‐rerupture BBAs. Hence, we speculate M2 macrophages participated in the tissue repairment after initial BBA rupture, facilitating the tissue collagen synthesis and fibroblasts proliferation to occlude initial aneurysm breach. Nonetheless, this process was impaired by the reduced M2 macrophages in reruptured BBAs, causing the less stable aneurysm structure and induces aneurysm rerupture. Importantly, this provides a novel insight into the pathology of BBAs that modulating the migration and polarization of M2 macrophages and its cellular interaction with fibroblasts might serve as a promising new target for biologic treatment in BBAs.^30^ Further in vitro studies are warranted to validate the findings in the current observations.

## 5 CONCLUSIONS

BBAs poses histopathological features of occult pseudoaneurysms or dissecting aneurysms. Reduced M2 macrophages and adventitia collagen dampen the structural integrity of BBAs and induce preoperative rerupture. Targeting M2‐polarized macrophages and its interplay with arterial fibroblasts may be a future therapeutic potential.

## CONFLICT OF INTEREST

The authors declare no conflict of interest.

## AUTHOR CONTRIBUTIONS

DK WEN contributed to drafting/revision of the manuscript for content, including medical writing for content; played major role in the acquisition of data; contributed to study concept or design; and contributed to analysis or interpretation of data. RQ Chen contributed to drafting/revision of the manuscript for content, including medical writing for content, and played major role in the acquisition of data. H Li played major role in the acquisition of data. J Zheng contributed to drafting/revision of the manuscript for content, including medical writing for content, and played major role in the acquisition of data. Fu Wei played major role in the acquisition of data and analysis of data. ZY Shi played major role in the acquisition of data. C You contributed to drafting/revision of the manuscript for content, including medical writing for content, played major role in the acquisition of data. M Yang played major role in the acquisition of data; study concept; or design. L Ma contributed to drafting/revision of the manuscript for content, including medical writing for content; played major role in the acquisition of data; study concept or design; and analysis or interpretation of data.

## DISCLOSURES

None.

## Supporting information

Table S1‐S2

## Data Availability

The data that support the findings of this study are available on request from the corresponding author. The data are not publicly available due to privacy or ethical restrictions.
