# Supplementary material for: Reduced M2 macrophages and adventitia collagen dampen the structural integrity of blood blister–like aneurysms and induce preoperative rerupture
Source: Cell Prolif. 2021 Dec 30;55(2):e13175. doi: 10.1111/cpr.13175 (PMC8828257; doi:10.1111/cpr.13175)
Supplement: Supplementary file 1 — Table S1‐S2 [file CPR-55-e13175-s001.docx]

**Supplementary Table 1. Baseline information of saccular aneurysms and blood blister-like aneurysms samples.**

**Supplementary Table 2. Antibodies used in the assays.**
